# Evaluation of the anti-diarrheal effects of the whole plant extracts of *Cuscuta reflexa* Roxb in pigeons

**DOI:** 10.1016/j.toxrep.2021.02.013

**Published:** 2021-02-23

**Authors:** Naveed Muhammad, Sana Ullah, Abdur Rauf, Muhammad Atif, Seema Patel, Muhammad Israr, Sajid Akbar, Omer Shehzad, Muhammad Saeed, Saud Bawazeer, Md. Sahab Uddin, Marina Derkho, Mohammad Ali Shariati, Mohammad S. Mubarak

**Affiliations:** a Department of Pharmacy, Abdul Wali Khan University, Mardan, KPK, Pakistan; b Department of Chemistry, University of Swabi, Anbar, 23561, KPK, Pakistan; c Bioinformatics and Medical Informatics Research Center, San Diego State University, San Diego, 92182, USA; d Pakistan Science, Foundation, Islamabad, Islamabad, Pakistan; e Abbotabad University of Science and Technology, Abbotabad, KPK, Pakistan; f Department of Pharmacy, University of Peshawar, Peshawar, KPK, Pakistan; g Department of Pharmaceutical Chemistry, Faculty of Pharmacy, Umm Al-Qura University, Makkah, P.O. Box 42, Saudi Arabia; h Department of Pharmacy, Southeast University, Dhaka, Bangladesh; i Pharmakon Neuroscience Research Network, Dhaka, Bangladesh; j South-Ural State Agrarian University, Troitsk, Russian Federation; k K.G. Razumovsky Moscow State University of Technologies and Management (the First Cossack University), Moscow, Russian Federation; l Department of Chemistry, The University of Jordan, Amman, 11942, Jordan

**Keywords:** JCR, Juice of *Cuscuta reflexa*, CRAE, *Cuscuta reflexa* aqueous extract, CRME, *Cuscuta reflexa* methanolic extract, ANOVA, Analysis of variance, IM, Intra Muscular, PI, Percent inhibition, IV, Intravenously, *Cuscuta reflexa*, Antidiarrheal, Charcoal, castor oil, Magnesium sulfate

## Abstract

**Background:**

*Cuscuta reflexa* (dodder) belonging to the family Convolvulaceae has many ethno-medicinal uses such as antidiarrheal and antiemetic. This plant has been employed to treat diarrhea, where the antidiarrheal use of this plant is well established in different communities around the world without scientific bases. In addition, the antibacterial, anthelmintic, anticholinergic, and antihistaminic effects of this parasitic vine are partly responsible for the folkloric antidiarrheal use of this plant. In the present study, the antidiarrheal activity *of C. reflexa* was evaluated in pigeons (*Columba livia*) using the juice (JCR), aqueous (CRAE), and methanol (CRME) extracts.

**Methods:**

The antidiarrheal effect of *C. reflexa* was evaluated using different reported research models, with few modifications. In pigeons, diarrhea was induced by administration of castor oil (6 mL/kg, PO), ampicillin (250 mg/kg, IP), magnesium sulfate (2 gm/kg, PO), and cisplatin (6 mg/kg, IV). In these experiments, loperamide (2 mg/kg, IM) was used as a positive control, whereas JCR (1 mL/kg (1%) and 1 mL/kg (2%), CRAE (50, 100 and 200 mg/kg) and CRME (50, 100 and 200 mg/kg) were administered intramuscularly at different doses into each pigeon in the test groups.

**Results:**

In addition to cisplatin-induced diarrhea, all paradigms tested gave significant results (*P* < 0.01). The JCR, at different doses, exhibited a significant (*p* < 0.01) a dose-dependent antidiarrheal effect on both the frequency and the onset of diarrhea. Similarly, CRAE and CRME, at doses of 100 and 200 mg/kg, showed considerable (*p* < 0.001) inhibition against the onset and frequency of diarrhea. On the other hand, JCR, CRAE, and CRME exerted significant effects (*p* < 0.001) on the percentage inhibition (PI) of diarrhea and gastrointestinal charcoal transit in a dose-dependent manner. In this respect, the maximum PI (*p* < 0.01) of JCR, CRAE, and CRME in different experimental paradigms was 43.13, 49.14, and 55.99 %, respectively.

**Conclusions:**

Taken all together, results from this study reveal that the juice, aqueous, and methanol extract of *C. reflexa* exhibit significant anti-motility and anti-secretory potential. These findings may explain the medicinal use of *C. reflexa in* folk medicine as an antidiarrheal medicinal plant.

## Highlights

• The antidiarrheal activity of *C. reflexa* was evaluated in pigeons using the juice, aqueous, and methanol extracts.
• The antidiarrheal effect of *C. reflexa* was evaluated using different reported research models.
• The juice, aqueous, and methanol extract of *C. reflexa* exhibit significant anti-motility and anti-secretory potential.

## Graphical abstract

**Figure:**
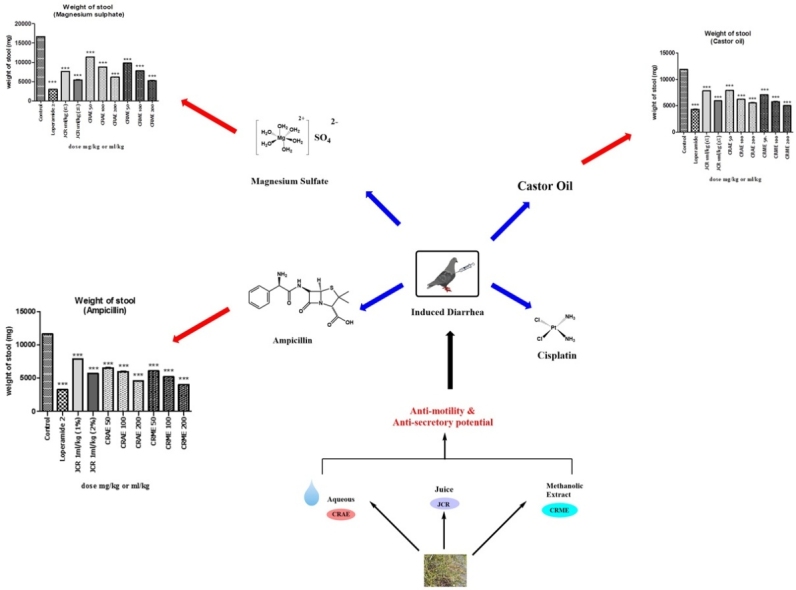

## 1 Introduction

Herbal remedies are used in both developed and undeveloped countries around the world, with several medicinal plants proving new therapeutic sources [1]. The World Health Organization (WHO) estimates that 90 % of the population in developing countries use herbal medicines [2]. The ethnobotanical literature is rich with information on herbal products as a remedy for chronic and acute disorders, such as constipation, vomiting, diarrhea, and antispasmodics, among others [3]. Interest in medicinal plants is growing, due to their increasing importance as medicinal sources [3]. Within this context, *C. reflexa*, belonging to the family *Convolvulaceae*, is a perennial parasitic herb found all over the world, including India, Bangladesh, Pakistan, and Nepal [2]. Several species of the genus *Cuscuta* also grow in the United States. The genus *Cuscuta* has 145 species, 14 of which thrive in Pakistan [4]. Plants of this family are leafless threads, like weeds, and are obligate parasites. Most species of this family are entirely dependent on the host plant [6]. Flowers of the vine are bell-shaped, white, and small with fleshy calyxes directly attached to the nodes of the stem [7].

*C. reflexa* is commonly called zailay or zara parwathye (Pushto), dodder (English), and akash bel (urdu) [8,9]. Its flowering season is from March to April, and is propagated by seeds [9]. Almost all parts of the plant such as the stem, fruits, seeds, and flowers are used as medicines [9]. Phytochemical analysis of *C. reflexa* shows that it contains mainly caffeic acid, flavonoids, tannins, and some phenolic components [10]. Other phytoconstituents isolated from *C. reflexa* are mannitol, dulcitol, carotenoids, quercetin-3-o-glucoside, sitosterol, myricetin, kaempferol, isorhametine-3−0-neohesperidoside, lycopene, bergenin, apigenin-7-β-rutinoside, lutein, kaempferol-3-o- glucoside, 6-hydroxy-4-(4-hydroxypheny1)-7-methoxy coumarin, hyperoside, quercetin, 6,7-dimethoxy coumarin (scoparone), α-amyrin, kaempferol-3−0-α-rhamnoside, apigenin-7-O-glucoside, myricetin-3−0-α-rhamnoside, coccinoside B, reflexin, lycopene, carotene,β-setosterol, stigmasterol, a-cryptoxanthin, amarbeline (pigment), palmitic acid, stearic acid, 3−0-Caffeoyl quinic acid, abscisic acid (leaves), leuteolin, phytosterols (seeds), cuscutin (stem), cuscutamine, quercetin, amino acids, cuscutoside-A, cuscutaline, quinic acid and 3,4−0-dicaffeoyl quinic acid [[10], [11], [12]].

In Pakistan, the plant is processed in the form of various herbal preparations for the treatment of nausea, vomiting, diarrhea, jaundice, blood purification, itching, epilepsy, lumbago, paralysis, persistent chronic fever, muscle pain, liver disorders, and joint pain, among others. In this regard, numerous herbal products, such as Bukharin, Aftimooni, Majoon Najah, and Majoon Ushbaare, are available on the Pakistani market and contain *C. reflexa* in various quantities, mostly as a tonic. *C. reflexa* is known as a miracle plant, which is why a large number of pharmacological studies have been conducted on it, using different research methodologies and experiments. These pharmacological studies indicate the use of this plant as an antifungal [13], antibacterial, to lower blood pressure [14], anti-inflammatory [15], antioxidant [13], for the treatment of alopecia [16], anti-HIV [17], anthelmintic [18], antihistamine [19], anticholinergic, antidiabetic [20], anticonvulsant [21], CNS depression [22], muscle relaxant, analgesic, antitumor [23], and diuretic [24], among others.

A number of published articles have reported the antidiarrheal uses of *C. reflexa* by different communities, although experimental evidence is lacking [[25], [26], [27]]. *C. reflexa* stem’s juice is commonly fed to cattle three times a day, to treat diarrhea [28]. The validated anticholinergic [29], antihistamine, antifungal, and antibacterial [9] effects of this plant suggest further scientific studies on its antidiarrheal effect. Based on the above discussion, the objective of the present study was to evaluate the antidiarrheal effect of *C. reflexa* plant.

## 2 Materials and methods

### 2.1 Plant material

*C. reflexa*, which grows on the host plant *Eucalyptus globulus*, was collected in March 2016 from Takhti-i-Bahi Mardan area, Khyber Pakhtunkhwa, Pakistan. Dr. Moheib Shah, a botanist at the Botany department, Abdul Wali Khan University, Mardan, KPK, Pakistan, identified and authenticated the plant. A voucher specimen AWKUM.BOT.189(1)2 was deposited at the herbarium located at the Botany department, Abdul Wali Khan University, Mardan, KPK, Pakistan. The plant sample was dried at room temperature and crushed into powder.

### 2.2 Drugs and chemicals

Chemicals used throughout this investigation, including distilled water (Unisa pharmaceuticals (PVT) LTD, Pakistan), ampicillin (Bosch Pharmaceuticals (PVT) LTD, Pakistan), magnesium sulfate (Unisa Pharmaceuticals (PVT) LTD, Pakistan), castor oil (Karachi Pharmaceuticals Laboratory), cisplatin (Pfizer Laboratories LTD), loperamide HCl (Janssen-Cliag Pharmaceuticals (PVT) LTD), activated charcoal (India), and chloroform (Pfizer Laboratories LTD) were obtained from commercial sources (in parentheses) and used as received without further purification.

### 2.3 Experimental models

Healthy pigeons (240–380 g) of either sex obtained from the Department of Animals at the University of Pharmacy in Peshawar were used through this investigation. These animals were housed, at ambient temperature (25–30 °C) and light/dark cycles for 12/12 h. They were given free access to standard diet (locally available food; millet + wheat grains) and water. All experimental measures were approved by the Ethics Committee of the Department of Pharmacy of the University of Peshawar, Peshawar, Pakistan. Research work was conducted in accordance with the internationally accepted principles for the use and care of laboratory animals [30].

### 2.4 Preparation of juice and extract

#### 2.4.1 Juice

Approximately 3.2 kg of whole plant of *C. reflexa*, was cleaned, dried, cut into smaller pieces, and then passed through a pressing mill. Approximately 70 mL of juice was obtained, which was stored in a transparent nonreactive plastic bottle for experimental use. The fresh juice was used within 10 days of pressing to ensure good results.

#### 2.4.2 Aqueous extract

Five kilograms of the whole plant of *C. reflexa*, was cleaned and dried in the shade at room temperature (30–35 °C), and was carefully powdered. The powder (3.2 kg) was then macerated with water (6 L) at room temperature for 15 days with occasional shaking and stirring. The water-soluble residue was filtered with a muslin cloth and then with filter paper (Whatman No. 1). This process was repeated with the addition of 3 L of water to the macerated powder. The filtrate was kept in the shade at room temperature for complete evaporation of the water, which took 40 days. Afterwards, 28 g of solid was obtained as a crude aqueous extract, which was then reduced to powder to facilitate its dissolution.

### 2.5 Methanol extract

The methanol extract of *C. reflexa* was a generous donation by Herbion Pakistan Pharmaceutical (PVT) Limited, one of the leading manufacturers of herbal products in Pakistan.

### 2.6 Preparation of sample for in vivo *administration*

#### 2.6.1 Juice

Juice samples (1 and 2% solution of the *C. reflexa*) were prepared by dissolving 1 and 2 mL of fresh juice in 100 mL of distilled water under vigorous agitation. Subsequently, the term JCR will be used for the juice. The resulting JCR was stored in the refrigerator for later analysis.

#### 2.6.2 Aqueous extract

Aqueous extract solution was prepared by dissolving crude extract powder in distilled water with continuous and vigorous stirring. Subsequently, the term CRAE will be used for the aqueous extract. Samples of prepared CRAE were kept in a refrigerator for further analysis.

#### 2.6.3 Methanol extract

Methanol extract solution was prepared by dissolving a crude extract in distilled water with vigorous stirring. Subsequently, the term CRME will be used for the methanol extract. CRME prepared sample was also stored in refrigerator for further study

### 2.7 Experimental design

#### 2.7.1 Grouping and dosing for antidiarrheal model

Birds were randomly divided into ten groups of six pigeons each (n = 6). All groups were starved for 6 h, and allowed free access to drinking water prior to experiments. Group 01 received distilled water (10 mL/kg, intramuscularly) and served as a negative control group. Group 02 was treated with loperamide HCl (2 mg/kg, IM), which served as a positive control group. Different doses of JCR 1 mL/kg (1%) and 1 mL/kg (2%) IM were administered to groups 03 and 04, respectively. Animals in groups 05–07 were treated with different doses of CRAE 50, 100, and 200 mg/kg, IM, respectively. CRMEs at doses of 50, 100, and 200 mg/kg IM were administered into groups 08–10, respectively [31]. Dose selection was made on the basis of experimental test prior to the start of the actual experiment. After 30 min of administration of the test samples, each animal received diarrheal agents. All birds tested were carefully observed according to a pre-established protocol with slight modifications, depending on the time of first bowel movement, total number of bowel movements (12 h), total number of loose stools (12 h), stool weight, and percent inhibition of diarrhea [32,33]. The percentage inhibition of diarrhea was calculated using the following formula for each diarrhea -induced drug [33].

$$\text{P}\text{e}\text{r}\text{c}\text{e}\text{n}\text{t}\;\text{i}\text{n}\text{h}\text{i}\text{b}\text{i}\text{t}\text{i}\text{o}\text{n}\;=\frac{\text{M}\text{e}\text{a}\text{n}\;\text{n}\text{u}\text{m}\text{b}\text{e}\text{r}\;\text{o}\text{f}\;\text{w}\text{a}\text{t}\text{e}\text{r}\text{y}\;\text{s}\text{t}\text{o}\text{o}\text{l}\text{s}\;\left(\text{n}\text{e}\text{g}\text{a}\text{t}\text{i}\text{v}\text{e}\;\text{g}\text{r}\text{o}\text{u}\text{p}-\;\text{t}\text{r}\text{e}\text{a}\text{t}\text{e}\text{d}\;\text{g}\text{r}\text{o}\text{u}\text{p}\right)}{\text{M}\text{e}\text{a}\text{n}\;\text{n}\text{u}\text{m}\text{b}\text{e}\text{r}\;\text{o}\text{f}\;\text{w}\text{a}\text{t}\text{e}\text{r}\text{y}\;\text{s}\text{t}\text{o}\text{o}\text{l}\text{s}\;\text{o}\text{f}\;\text{n}\text{e}\text{g}\text{a}\text{t}\text{i}\text{v}\text{e}\;\text{g}\text{r}\text{o}\text{u}\text{p}}\times \;100$$

These results were compared with the negative and positive groups to assess antidiarrheal activity.

#### 2.7.2 Grouping and dosing for gastrointestinal transit model

For the charcoal meal GI transit test experiment, pigeons were divided into different groups of six birds each (n = 6). The group administered with distilled water (10 mL/mg, IM) served as the negative group. The group fed with loperamide (2 mg/kg, IM) acted as a positive group. The rest of the groups were treated with different doses of JCR, CRAE, and CRME, as described above. After 30 min, each animal in a group received a charcoal meal (8 mL/kg, PO), as suspension in distilled water, containing 5% acacia gum and 10 % vegetable charcoal. Pigeons were sacrificed by cervical dislocation after 30 min of treatment with the charcoal meal. Their abdomens were opened and their small intestines were dissected [19,25]. The distance travelled by the charcoal was measured and the peristaltic index was calculated in all euthanized birds using the following formula [33].

$$\text{P}\text{e}\text{r}\text{i}\text{s}\text{t}\text{a}\text{l}\text{t}\text{i}\text{c}\;\text{i}\text{n}\text{d}\text{e}\text{x}\;=\frac{\text{M}\text{e}\text{a}\text{n}\;\text{d}\text{i}\text{s}\text{t}\text{a}\text{n}\text{c}\text{e}\;\text{t}\text{r}\text{a}\text{v}\text{e}\text{l}\text{l}\text{e}\text{d}\;\text{b}\text{y}\;\text{c}\text{h}\text{a}\text{r}\text{c}\text{o}\text{a}\text{l}\;\text{t}\text{h}\text{r}\text{o}\text{u}\text{g}\text{h}\;\text{s}\text{m}\text{a}\text{l}\text{l}\;\text{i}\text{n}\text{t}\text{e}\text{s}\text{t}\text{i}\text{n}\text{e}}{\text{T}\text{o}\text{t}\text{a}\text{l}\;\text{l}\text{e}\text{n}\text{g}\text{t}\text{h}\;\text{o}\text{f}\;\text{s}\text{m}\text{a}\text{l}\text{l}\;\text{i}\text{n}\text{t}\text{e}\text{s}\text{t}\text{i}\text{n}\text{e}}\times \;100$$

The percentage inhibition (% inhibition) relative to the negative control group was also calculated using the following formula [33].

$$\text{\%}\;\text{i}\text{n}\text{h}\text{i}\text{b}\text{i}\text{t}\text{i}\text{o}\text{n}\;\;=\frac{\text{M}\text{e}\text{a}\text{n}\;\text{d}\text{i}\text{s}\text{t}\text{a}\text{n}\text{c}\text{e}\;\text{t}\text{r}\text{a}\text{v}\text{e}\text{l}\text{l}\text{e}\text{d}\;\text{b}\text{y}\;\text{c}\text{h}\text{a}\text{r}\text{c}\text{o}\text{a}\text{l}(\text{n}\text{e}\text{g}\text{a}\text{t}\text{i}\text{v}\text{e}\;\text{g}\text{r}\text{o}\text{u}\text{p}\;–\;\text{t}\text{r}\text{e}\text{a}\text{t}\text{e}\text{d}\;\text{g}\text{r}\text{o}\text{u}\text{p})}{\text{M}\text{e}\text{a}\text{n}\;\text{d}\text{i}\text{s}\text{t}\text{a}\text{n}\text{c}\text{e}\;\text{t}\text{r}\text{a}\text{v}\text{e}\text{l}\text{l}\text{e}\text{d}\;\text{b}\text{y}\;\text{c}\text{h}\text{a}\text{r}\text{c}\text{o}\text{a}\text{l}\;\text{o}\text{f}\;\text{n}\text{e}\text{g}\text{a}\text{t}\text{i}\text{v}\text{e}\;\text{g}\text{r}\text{o}\text{u}\text{p}}\times \;100$$

### 2.8 Antidiarrheal activity

#### 2.8.1 Ampicillin-induced diarrheal model

The experiment was carried out according to a published procedure with slight modifications [34]. After 30 min of group treatment with distilled water, a standard drug, JCR, CRAE, and CRME, respectively, diarrhea was induced with ampicillin at a dose of 250 mg/kg, administered intraperitoneally. Pigeons were then observed for the number and reliability of fecal contents.

#### 2.8.2 Castor oil-induced diarrhea model

Animals, divided into specific groups, were treated with distilled water, a standard drug, JCR, CRAE, and CRME. After 30 min of the above treatment, castor oil at a dose of 6 mL/kg orally was administered into each animal for induction of diarrhea. Pigeons were carefully observed for the number and consistency of fecal contents and compared with the negative group.

#### 2.8.3 Magnesium sulfate-induced diarrhea model

This experiment was carried out according to a method described above, with slight modifications [35]. Test animals were treated with distilled water, a standard drug, JCR, CRAE, and CRME. After 30 min of this treatment, diarrhea was induced with magnesium sulfate at a dose of 2 gm/kg orally.

#### 2.8.4 Cisplatin-induced diarrhea model

Animals were treated with distilled water, a standard drug, JCR, CRAE, and CRME. After 30 min of this treatment, cisplatin was administered intravenously into each animal at a dose of 6 mg/kg for the induction of diarrhea [29]. Pigeons were carefully observed with regard to the number and consistency of the fecal content and were compared with the negative group.

#### 2.8.5 Charcoal gastrointestinal transit model

For charcoal gastrointestinal transit assay, model animals were divided into specific groups and treated with different doses of distilled water, standard drug and tested samples (JCR, CRAE and CRME). After 30 min, each animal received a charcoal meal (8 mL/kg, PO). Animals were sacrificed by cervical dislocation and small intestines were removed [25].

### 2.9 Statistical analysis

GraphPad Prism (GraphPad software, San Diego, California, USA) was used for statistical analysis. Data are expressed as the mean ± standard error of the mean (SEM) and the median of the useful concentrations with 95 % confidence intervals. A unidirectional analysis of variance (ANOVA) followed by the Dunnett’s test was used to assess the antidiarrheal activity.

## 3 Results

### 3.1 Ampicillin-induced diarrhea

**Fig. 1:**
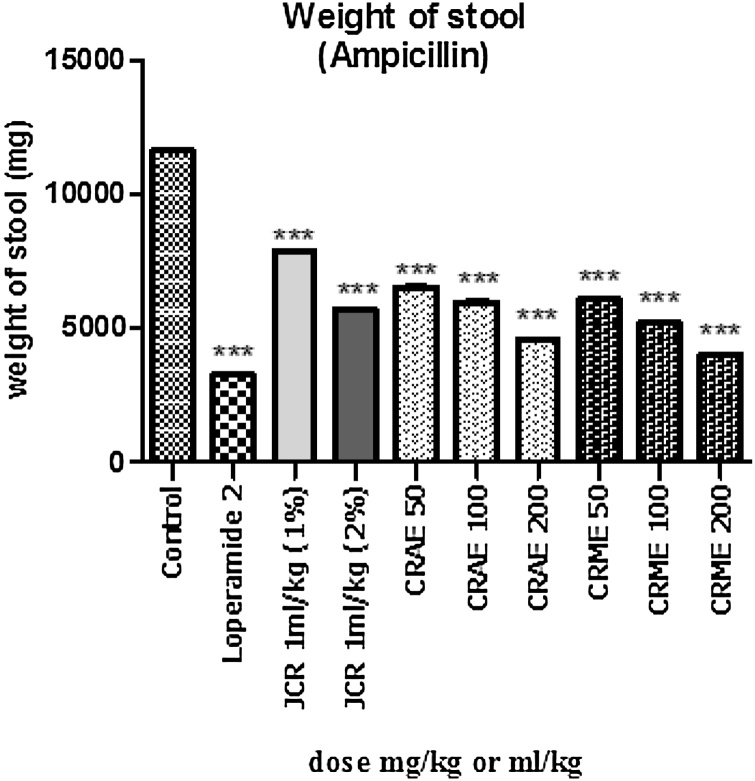
In the ampicillin-induced diarrhea, the weight of stool after various interventions was recorded.

After pre-treatment of pigeons with test plant material and then with ampicillin (250 mg/kg, IP) for the induction of diarrhea, a dose-dependent antidiarrheal potential was observed (Fig. 1).

#### 3.1.1 Effect of JCR

Results from our investigation showed that JCR at 1 mL/kg (2%) exhibits significant results (*p* < 0.001) for 1 st stool time, mean number of stools, and mean number of aqueous stools compared to the negative control as displayed in Table 1. Significant attenuation (*p* < 0.001) of stool weight reduction was observed for JCR at both concentrations. Additionally, results indicated that the percentage inhibition of diarrhea by JCR relative to the control was 32.57 and 40.24 %, respectively, while an inhibition of 71.46 % was established for the loperamide (2 mg/kg)-treated group.

**Table 1:** Effect of JCR, CRAE and CRME on ampicillin-induced diarrhea in pigeons.

Group (mg/kg, IM)	1^st^ stool time (min)	Mean no. of stools (8 h)	Mean no. of watery stools (8 h)	Percent inhibition (%)
Control	8.23 ± 2.83	9.42 ± 2.24	7.43 ± 1.37	0.00
Loperamide 2	19.66 ± 2.36***	5.65 ± 1.37**	2.12 ± 0.57**	71.46
JCR 1 mL/kg (1%)	10.42 ± 2.14^ns^	8.12 ± 1.57^ns^	5.01 ± 0.95^ns^	32.57
JCR 1 mL/kg (2%)	13.67 ± 4.27***	7.23 ± 1.86*	4.44 ± 1.14**	40.24
CRAE 50	9.38 ± 3.96^ns^	8.45 ± 1.57^ns^	6.12 ± 2.91^ns^	17.63
CRAE 100	11.76 ± 4.23*	7.51 ± 1.83*	5.67 ± 1.14*	23.68
CRAE200	13.44 ± 2.79**	7.04 ± 1.44*	4.69 ± 1.40*	36.87
CRME 50	10.88 ± 2.03^ns^	7.97 ± 0.57^ns^	5.98 ± 0.96^ns^	19.51
CRME 100	12.65 ± 3.26**	7.14 ± 2.88*	5.14 ± 1.21*	30.82
CRME 200	14.84 ± 3.72***	6.95 ± 1.15**	4.21 ± 1.65**	43.33

*Data are presented as the mean ± standard error of the mean for groups (n = 6). A One-way ANOVA followed by Dunnett’s test was applied for data analysis. *p < 0.05, **p < 0.01, ***p < 0.001 versus the negative group (Asterisks representing statistically significant values from control).

#### 3.1.2 Effect of CEAE

Our findings indicated that CRAE at 100 mg/kg shows significant (*p* < 0.05) effect for time to first bowel movement, mean number of bowel movements, and mean number of loose stools (Table 1). On the other hand, CRAE at the higher dose of 200 mg/kg significantly (*p* < 0.001) attenuated first bowel movement time, mean number of bowel movements, and mean number of loose stools compared to the negative group. CRAE at the 50 mg/kg dose did not cause significant effects; however, it is interesting to note that CRAE at all doses significantly (*p* < 0.001) reduced stool weight. The percentage inhibition of diarrhea by CRAE at 50, 100, and 200 mg/kg was 17.63, 23.68, and 36.68 %, respectively, compared to the negative group.

#### 3.1.3 Effect of CRME

At the 100 mg/kg dose, CRME showed significant (*p* < 0.01) effect with respect to stool time, mean number of stools, and mean number of loose stools compared to the negative group, as shown in Table 1. CRME at the 200 mg/kg dose exhibited significant (*p* < 0.001) effects for all test parameters. CRME at 100 and 200 mg/kg produced a significant reduction in stool weight. The percentage inhibition of diarrhea relative to the negative group was 19.51, 30.82, and 43.33 % at 50, 100, and 200 mg/kg, respectively.

### 3.2 Castor oil-induced diarrhea

**Fig. 2:**
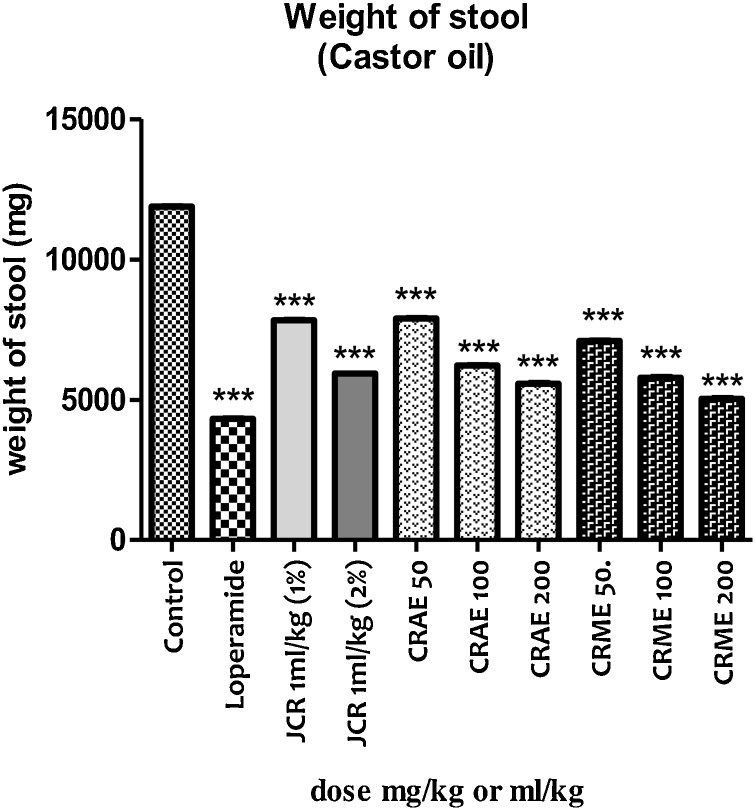
In the castor oil-induced diarrhea, the weight of stool after various interventions was recorded.

Tested samples of *C. reflexa* showed a dose-dependent antidiarrheal effect against castor oil-induced diarrhea (Fig. 2).

#### 3.2.1 Effect of JCR

Results from our study revealed that JCR at the dose of 1 mL/kg (1%) did not show any significant effect regarding all aspects. However, it exhibited significant (*p* < 0.001) less weight of stool as given in Table 2. At a higher dose of 1 mL/kg (2%), JCR significantly (*p* < 0.01) attenuated the 1st stool time, mean number of stools, and mean number of watery stools when compared to the negative group. At this later dose, JCR caused considerable (*p* < 0.001) less weight of stool. Percentage inhibition of diarrhea by JCR at the dose of 1 mL/kg (1%) and 1 mL/kg (2%) was 14.19 and 43.13 %, respectively, while the percent inhibition caused by the standard drug (loperamide) was 75.60 %.

**Table 2:** Effect of JCR, CRAE, and CRME on castor oil-induced diarrhea in pigeons.

Group (mg/kg, IM)	1^st^ stool time (min)	Mean no. of stools (8 h)	Mean no. of watery stools (8 h)	Percent inhibition (%)
Control	13.11 ± 2.57	15.18 ± 2.28	11.15 ± 3.40	0.00
Loperamide, 2	26.65 ± 3.86***	6.42 ± 2.57***	2.72 ± 0.63***	75.60
JCR 1 mL/kg (1%)	15.20 ± 2.34^ns^	12.45 ± 2.13^ns^	9.48 ± 1.38^ns^	14.97
JCR 1 mL/kg (2%)	20.18 ± 3.65**	10.83 ± 2.35*	6.34 ± 1.54*	43.13
CRAE 50	15.87 ± 3.42^ns^	12.87 ± 2.01^ns^	8.62 ± 1.14^ns^	22.69
CRAE 100	18.93 ± 2.65^ns^	10.70 ± 2.34^ns^	7.30 ± 1.21^ns^	34.52
CRAE200	21.42 ± 4.85**	9.72 ± 2.66*	5.67 ± 1.47*	49.14
CRME 50	16.91 ± 3.49^ns^	11.71 ± 3.13^ns^	8.01 ± 1.16^ns^	28.16
CRME 100	20.65 ± 4.68*	10.01 ± 2.28*	6.88 ± 1.28*	38.29
CRME 200	22.64 ± 4.92**	8.96 ± 1.72**	4.93 ± 1.53**	55.84

*Data are presented as the mean ± standard error of the mean for groups (n = 6). A One-way ANOVA followed by Dunnett’s test was applied for data analysis. *p < 0.05, ** *p* < 0.01, ***p < 0.001 versus the negative group (Asterisks representing statistically significant values from control).

#### 3.2.2 Effect of CRAE

The CRAE at 50 and 100 mg/kg was almost invalid, but stool weight results were highly significant (*p* < 0.001) as clearly shown in Table 2. The CRAE at 200 mg/kg showed significant results (*p* < 0.01) with respect to time to first bowel movement, mean number of bowel movements, and mean number of loose stools compared to the control group. The percentage inhibition of diarrhea relative to the control group was 22.69, 34.52, and 49.14 % at 50, 100 and 200 mg/kg, respectively.

#### 3.2.3 Effect of CRME

CRME at the dose of 50 mg/kg was non-significant when compared to the negative group (Table 2). At the dose of 100 mg/kg, CRME significantly attenuated (*p* < 0.5) 1st stool time, mean number of stools, and mean number of watery stools, as compared to the negative group. CRME at the dose of 200 mg/kg, exhibited significant (*p* < 0.01) results relative to 1st stool time, mean number of stools, and mean number of watery stools. CRME at the dose of 50, 100 and 200 mg/kg produced notably (*p* < 0.001) less weight of stool as compared to the distilled water treated group. The percent inhibition of diarrhea by CRME at the dose of 50, 100, and 200 mg/kg was 28.16, 38.28, and 55.84 %, respectively.

CRME at 50 mg/kg was insignificant compared to the negative group (Table 2). At the 100 mg/kg dose, CRME significantly (*p* < 0.5) reduced (*p* < 0.5) the time to first bowel movement, mean number of bowel movements, and mean number of loose stools compared to the negative group. At the 200 mg/kg dose, CRME showed significant (*p* < 0.01) results with respect to time to the first bowel movement, mean number of bowel movements, and mean number of loose stools. CRME at 50, 100 and 200 mg/kg produced significantly lower stool weights (*p* < 0.001) compared to the distilled water group. The percentage inhibition of diarrhea by CRME at 50, 100, and 200 mg/kg was 28.16, 38.28 and 55.84 %, respectively.

### 3.3 Magnesium sulfate-induced diarrhea

**Fig. 3:**
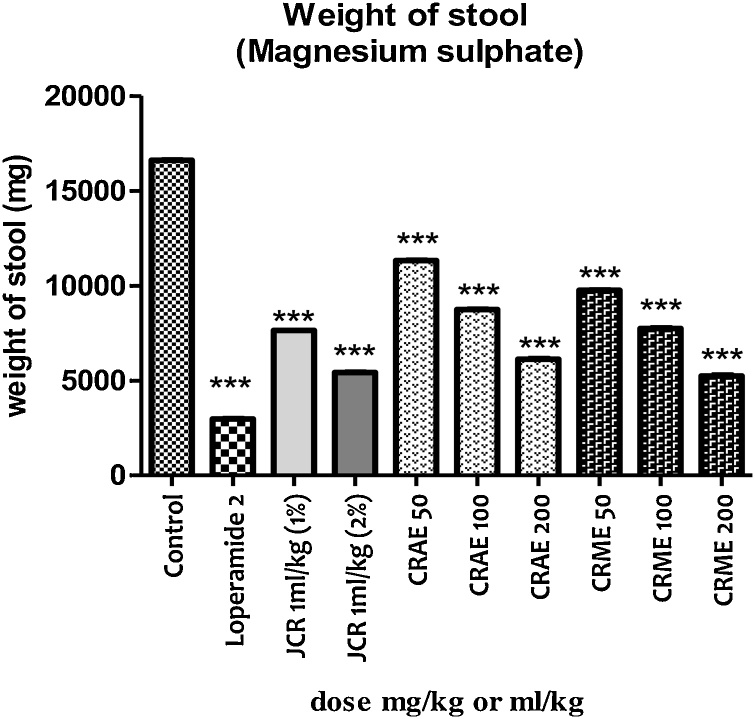
In the magnesium sulfate-induced diarrhea, the weight of stool after various interventions was recorded.

Pretreatment of pigeons with different doses of JCR, CRAE, and CRME caused a concentration-dependent antidiarrheal effect against magnesium sulfate-induced diarrhea (Fig. 3).

#### 3.3.1 Effect of JCR

The JCR at the 1 mL/kg (1%) dose did not cause any significant effect as shown in Table 3, however, at this dose results for stool weight were significant (*p* < 0.001) compared to the distilled water group. At the higher dose of 1 mL/kg (2%), JCR reduced the time to first stool, mean number of stools, and mean number of aqueous stools significantly (*p* < 0.05), while stool weight results were remarkable (*p* < 0.001). Results also showed that the percentage inhibition of diarrhea compared to JCR at 1 mL/kg (1%), 1 mL/kg (2%), and loperamide were 33.17, 40.58, and 76.60 % respectively, compared to the negative group.

**Table 3:** Effect of JCR, CRAE and CRME against magnesium sulfate-induced diarrhea in pigeons.

Group (mg/kg, IM)	1^st^ stool time (min)	Mean no. of stools (8 h)	Mean no. of watery stools (8 h)	Percent inhibition (%)
Control	10.12 ± 2.57	16.56 ± 3.40	12.27 ± 3.15	0.00
Loperamide 2	24.42 ± 3.15***	7.23 ± 2.57***	2.87 ± 0.50***	76.60
JCR 1 mL/kg (1%)	13.96 ± 2.73^ns^	12.38 ± 2.73^ns^	8.20 ± 2.15^ns^	33.17
JCR 1 mL/kg (2%)	17.88 ± 4.30*	10.04 ± 2.30*	7.29 ± 2.73*	40.58
CRAE 50	12.65 ± 3.53^ns^	14.35 ± 1.04^ns^	9.65 ± 2.77^ns^	21.35
CRAE 100	15.36 ± 2.89^ns^	12.81 ± 2.21^ns^	6.35 ± 1.08*	48.24
CRAE200	17.98 ± 2.24*	11.12 ± 2.53*	5.83 ± 1.28**	52.48
CRME 50	12.99 ± 2.72^ns^	13.53 ± 3.08^ns^	8.71 ± 1.91^ns^	29.01
CRME 100	16.81 ± 3.06*	11.25 ± 3.28*	6.01 ± 1.14**	51.01
CRME 200	18.53 ± 3.30**	10.25 ± 2.61**	5.40 ± 1.46***	55.99

*Data are presented as the mean ± standard error of the mean for groups (n = 6). A One-way ANOVA followed by Dunnett’s test was applied for data analysis. *p < 0.05, **p < 0.01, ***p < 0.001 versus negative group (Asterisks representing statistically significant values from control).

#### 3.3.2 Effect of CRAE

At the 50 mg/kg dose, RCT was almost insignificant compared to the distilled water group; however, in terms of the mean stool weight, results were significant (*p* < 0.001) as shown in Table 3. Additionally, CRAE at the 100 mg/kg dose did not show much effect with respect to time to the first stool and mean number of stools, but caused significant influence with respect to the mean number of aqueous stools (*p* < 0.05). On the other hand, CRAE at the highest dose (200 mg/kg), exhibited significant (*p* < 0.01) effect with respect to first stool time, mean number of stools, and mean number of liquid stools. However, it is interesting to note that CRAE at the 100 and 200 mg/kg dose showed significant lower mean stool weight (*p* < 0.001) compared to the group treated with distilled water. The percentage inhibition of diarrhea compared to the CRAE at 50, 100, and 200 mg/kg was 21.35, 48.24 and 52.48 %, respectively.

#### 3.3.3 Effect of CRME

Results related to the effect of CRME showed that at 50 mg/kg, the extract produced significant (*p* < 0.001) lower mean stool weights compared to the negative control group as shown in Table 3. However, at the 100 mg/kg dose, results were promising (*p* < 0.01) in terms of time to first bowel movement, mean number of bowel movements, and mean number of loose stools. Similarly, CRME at 200 mg/kg was highly significant (*p* < 0.001) for time to first bowel movement, mean number of bowel movements, and mean number of loose stools. With respect to stool weight, the CRME at the 100 and 200 mg/kg dose showed a significantly (*p* < 0.001) lower weight (*p* < 0.001) compared to the group treated with distilled water. The percentage inhibition of diarrhea by CRME at 50, 100, and 200 mg/kg was 29.01, 51.01, and 55.99 %, respectively compared to the negative control group.

### 3.4 Cisplatin-induced diarrhea

Results of our investigation demonstrated that pre-treatment of pigeons with JCR, CRAE, and CRME at different doses did not show significant antidiarrheal effects the cisplatin-induced diarrheal group.

#### 3.4.1 Effect of JCR

JCR at the 1 mL/kg (1%) and 1 mL/kg (2%) dose did not show antidiarrheal effects in any aspect; however, at higher doses, JCR caused significantly lower stool weight (p < 0.001) compared to the group treated with distilled water as shown in Table 4. JCR relative to the percentage inhibition of diarrhea also did not show significant results at its lower dose, but at the higher dose of 1 mg/kg (2%), the percentage inhibition of diarrhea was 16.04 % as compare to that of loperamide (43.58 % relative to the negative group).

**Table 4:** Effect of JCR, CRAE and CRME on cisplatin-induced diarrhea in pigeons.

Group (mg/kg, IM)	1^st^ stool time (min)	Total number of tools (mean)	Total number of watery stools (mean)		Percent inhibition (%)
Control	23.32 ± 3.03	19.33 ± 2.75		18.63 ± 3.36	0.00
Loperamide 2	44.98 ± 5.15***	11.61 ± 3.92**		10.51 ± 2.57***	43.58
JCR1 ml/kg 1%)	18.98 ± 3.59^ns^	19.43 ± 2.43^ns^		18.71 ± 2.21^ns^	−0.42
JCR1 ml/kg(2%)	28.79 ± 4.98^ns^	18.64 ± 4.71^ns^		15.64 ± 3.73^ns^	16.04
CRAE 50	25.87 ± 4.54^ns^	27.27 ± 3.34^ns^		20.58 ± 3.02^ns^	−10.46
CRAE 100	29.39 ± 3.99^ns^	25.35 ± 2.96^ns^		17.34 ± 3.64^ns^	6.92
CRAE200	27.98 ± 4.72^ns^	18.99 ± 3.66^ns^		14.35 ± 2.34^ns^	22.97
CRME 50	28.93 ± 4.85^ns^	23.36 ± 3.72^ns^		21.87 ± 4.47^ns^	−17.39
CRME 100	26.97 ± 4.59^ns^	17.39 ± 3.40^ns^		16.39 ± 3.14^ns^	12.02
CRME 200	30.81 ± 2.11*	20.25 ± 4.11^ns^		15.46 ± 3.79^ns^	17.01

*Data are presented as mean ± standard error of the mean for groups (n = 6). A One-way ANOVA followed by Dunnett’s test was applied for data analysis. *p < 0.05, **p < 0.01, ***p < 0.001 versus negative group (Asterisks representing statistically significant values from control).

#### 3.4.2 Effect of CRAE

Results showed that the CRAE at a dose of 50 mg/kg, 100 and 200 mg/kg, was not active, and were comparable to those of the distilled water group. However, it is interesting to note that at the 100 and 200 mg/kg doses, it significantly lowered the mean stool weight (*p* < 0.001) as shown in Table 4. In addition, results showed that the percentage inhibition of diarrhea relative to the CRAE at 50 mg/kg was also not significant, but at 100 and 200 mg/kg, the percentage inhibition was significant at 6.92 % and 22.97 %, respectively.

#### 3.4.3 Effect of CRME

CRME at 50 mg/kg, 100, and 200 mg/kg was almost insignificant as clearly shown in Table 4; however, at 100 and 200 mg/kg it produced significantly (*p* < 0.001) lower stool weights than the distilled water group. The percentage inhibition of diarrhea relative to CRME at 50 mg/kg did not show significant results; however, at 100 and 200 mg/kg, the percentage inhibition was 12.02 and 17.01 % respectively.

### 3.5 Charcoal gastrointestinal transit test

**Fig. 4:**
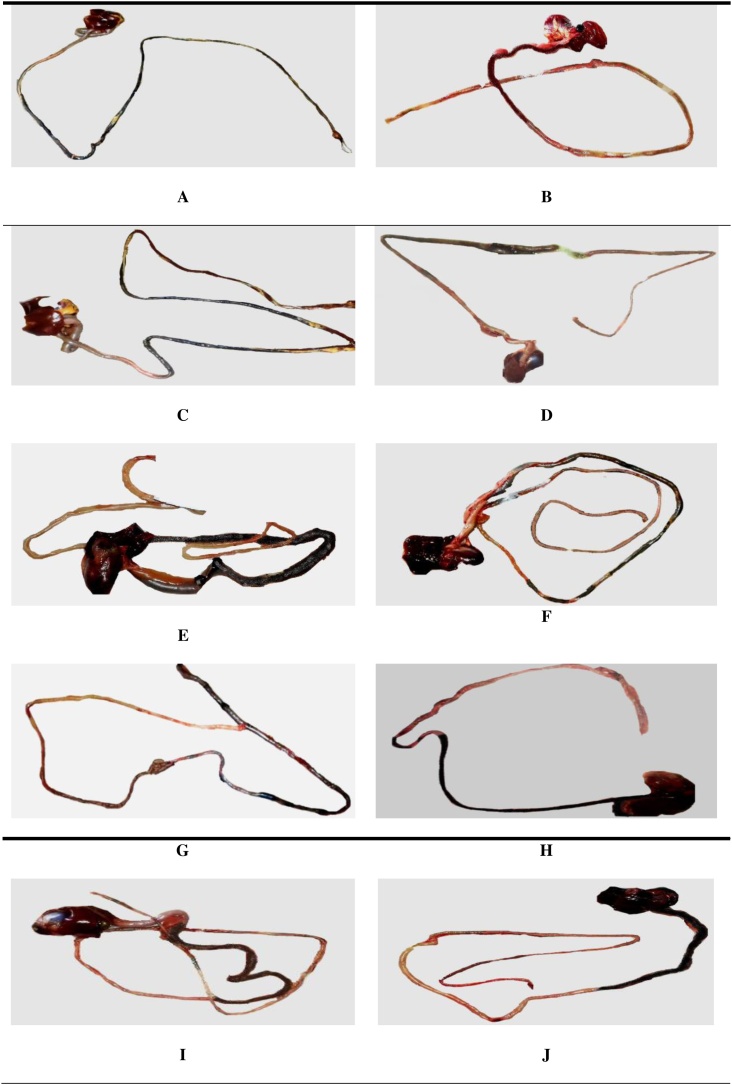
**A;** Small intestine of pigeon treated with distilled water, **B:** Small intestine of pigeon treated with the standard drug (Loperamide), **C:** Small intestine of pigeon treated with 1 mL/kg (1%) JCR, **D:** Small intestine of pigeon treated with 1 mL/kg (2%) JCR, **E:** Small intestine of pigeon treated with 50 mg/kg CRAE, **F:** Small intestine of pigeon treated with 100 mg/kg CRAE, **G:** Small intestine of pigeon treated with 200 mg/kg CREA, **H:** Small intestine of pigeon treated with 50 mg/kg CRME, **I:** Small intestine of pigeon treated with 100 mg/kg CRME, and **J:** Small intestine of pigeon treated with 200 mg/kg CRME.

Our results showed that JCR, CRAE, and CRME exhibit a significant normal dose-dependent propulsion of charcoal flour as depicted in Fig. 4.

#### 3.5.1 Effect of JCR

Results from this investigation revealed that JCR inhibits normal intestinal transit of charcoal plug by 22.47 and 41.42 % at the dose of 1 mL/kg (1%) and 1 mL/kg (2%), respectively, whereas inhibition caused by the standard (loperamide) drug was 57.46 % as shown in Table 5. On the other hand, the peristaltic index of JCR at the dose of 1 mL/kg (1%) and 1 mL/kg (2%) was 69.64 and 57.87, respectively, and was 40.48 for the standard drug.

**Table 5:** Effect of JCR, CRAE, and CRME on charcoal meal peristaltic index.

Group(mg/kg,IM)	Mean of total length of small intestine (inches)	Distance travelled by charcoal (inches)	Peristaltic index	Inhibition (%)
Control	31.12 ± 1.09	26.87 ± 0.502	86.34	0.0
Loperamide 2	28.23 ± 1.15^ns^	11.43 ± 0.64***	40.48	57.46
JCR 1 mL/kg (1%)	29.91 ± 1.64^ns^	20.83 ± 1.73*	69.64	22.47
JCR 1 mL/kg (2%)	30.65 ± 2.30^ns^	15.74 ± 1.15**	57.87	41.42
CRAE 50	31.56 ± 1.40	20.59 ± 1.08^ns^	74.74	23.37
CRAE 100	30.76 ± 1.01	17.56 ± 1.14***	60.33	34.64
CRAE 200	27.87 ± 1.08	13.67 ± 1.66***	56.22	49.12
CRME 50	29.74 ± 0.57	20.51 ± 0.87*	72.32	23.66
CRME 100	31.87 ± 1.08	16.91 ± 1.40***	59.33	37.06
CRME 200	28.65 ± 0.48	13.19 ± 1.34***	51.78	50.91

*Data are presented as the mean ± standard error of the mean for groups (n = 6). A One-way ANOVA followed by Dunnett’s test were applied for data analysis. *p < 0.05, **p < 0.01, ***p < 0.001 versus the negative group (Asterisks representing statistically significant values from control).

#### 3.5.2 Effect of CRAE

Our findings from this study showed that CRAE at 50, 100, and 200 mg/kg inhibits charcoal meal movement by 23.37, 34.64, and 49.12 %, respectively, compared to the negative control (Table 5). Similarly, the charcoal movement or peristaltic charcoal index in the small intestine by CRAE at 50, 100 and 200 mg/kg was 74.74, 60.33, and 56.22, respectively.

#### 3.5.3 Effect of CRME

CRME at 50, 100, and 200 mg/kg inhibited charcoal meal movement by 23.66, 37.06, and 50.91 % compared to the negative control (Table 5). The peristaltic index of charcoal through the small intestine by CRME at 50, 100, and 200 mg/kg was 72.32, 59.33, and 51.78, respectively.

## 4 Discussion

Diarrhea is one of the main side effects of various drugs with different mechanisms. Diarrhea can be defined as the abnormal passage of loose or watery stools associated with an increase in the frequency of defecation of at least three loose or watery stools in one day [36]. The etiologies of diarrhea induction are osmotic diarrhea, secretory diarrhea, inflammatory diarrhea, drug-induced diarrhea, and diarrhea of altered motility due to GIT. In this study, we evaluated the antidiarrheal potential of *C. reflexa*, since antidiarrheal effects of this plant have been reported using different research models. Our findings indicated that JCR, CRAE, and CRME show statistically significant dose-dependent inhibition of all diarrheal parameters including time to 1 st stool, mean number of stools, mean number of loose stools, and mean stool weight compared to the negative group. These results are consistent with the existing literature on antidiarrheal herbs, as *C. reflexa* causes a reduction in stool count, wet stool count, and decreased stool weight, and diarrhea [14,33,35].

**Fig. 5:**
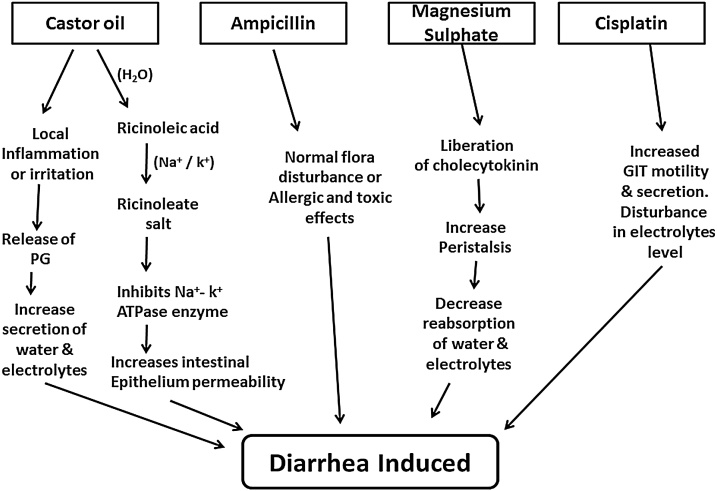
A schematic diagram of diarrhea induced by various agents.

Ampicillin can cause diarrhea by disrupting the number and function of normal intestinal flora, overgrowth of certain pathogens, affecting GIT motility, or by producing allergic and toxic effects. In this respect, castor oil has been used to induce diarrhea in various reported experiments. Castor oil induces diarrhea through the formation of ricinoleic acid from the hydrolysis of the oil. Ricinoleic acid reacts with the Na + and K + ions present in the lumen and forms ricinoleate salts. Ricinoleate salts inhibit the ATPase Na + and K + enzyme and cause significant changes in the water and electrolyte balance, thus increasing the permeability of the intestinal epithelium [33]. Local inflammation and irritation of the intestinal mucosa by ricinoleic acid have been reported. This acid causes the release of prostaglandin, which increases the net level of water and electrolyte secretion in the small intestine. Therefore, antidiarrheal drugs interfere with the motility or hypersecretion of GIT in the small intestine [19]. Magnesium sulfate (MgSO_4_) induces diarrhea in animals. It induces diarrhea by preventing the absorption of water and sodium chloride from the small intestine, which increases the intestinal contents of the animals and produces liquid stools (35). In addition, magnesium sulfate promotes the release of cholecytokinin from the duodenal mucosa, thereby increasing the secretion and motility of the small intestine (36). In a similar fashion, cisplatin is one of the most effective anti-cancer drugs worldwide. The most common side effects of cisplatin are nausea, vomiting, diarrhea, hypocalcemia, and nephrotoxicity, which reduce patient compliance [37]. The pathophysiology of cisplatin-induced diarrhea is extensive and complex, and may result from various mechanisms, such as secretion, malabsorption, osmotic, infection, inflammation, and changes in GIT motility [38]. The mechanisms of these diarrheal agents are shown in Fig. 5.

In general, the antidiarrheal effect of most herbs against experimentally induced diarrhea is caused by either an increase in fluid and electrolyte absorption or a slowing of intestinal transit, which allow more time for absorption [39]. Any effect of a medicinal plant such as an anticholinergic, antihistaminic effect, or inhibition of GIT secretion can lead to an antidiarrheal effect [40]. Interestingly, the presence of tannins and flavonoids in plants can lead to increased reabsorption of colon water and electrolytes [33]. In this respect, tannins in plants denature proteins in the intestinal mucosa, forming a protein-tannate complex, which in turn forms a layer on the intestinal mucosa, making it more resistant to chemical alterations and reduces secretions [41,42]. Similarly, flavonoids have antioxidant properties that are responsible for inhibiting certain enzymatic oxidations, including those involved in the metabolism of arachidonic acid [43], thereby reducing prostaglandin-induced liquid secretions. These compounds reduce both the weight and volume of the intraluminal contents, which is a direct consequence of the reduction of water and electrolyte secretion in the small intestine.

Our findings indicate that JCR, CRAE, and CRME inhibit gastrointestinal impulses in all diarrhea-induced experiments in a dose-dependent manner except in the cisplatin-induced paradigm. This antidiarrheal effect of *C. reflexa* might be attributed to its previously reported anticholinergic and antihistaminic effect, since both properties lead to relaxation of smooth muscle and GIT [44], thereby slowing GIT motility. A delay in GIT motility increases the retention of substances in the intestine and allows better absorption of water and electrolytes. This anticholinergic effect was also supported by tests of *C. reflexa* extract against the enzyme acetylcholinesterase. Stimulation of the cholinergic receptor causes smooth muscle contraction and increases mucus secretion in the airways and TIG [45]. It was also reported that stimulation of the cholinergic receptor has a direct effect on the circular muscle of the distal colon and causes massive contractions, thus facilitating the propulsion of fecal contents. In addition, Nausea, vomiting, diarrhea, bladder stimulation, sphincter relaxation, and the need to defecate have been reported in various publications in response to stimulation of muscarinic receptors by acetylcholine.

Published research on the mode of action of medicinal plants indicates that the presence of phytochemicals such as alkaloids, flavonoids, and terpenoids plays a role in inhibiting intestinal motility and electrolyte secretion. Findings also show that tannin-containing plants can reduce peristaltic movements and intestinal secretion by inhibiting the inward intracellular flow of Ca^2+^ or by activating the calcium pumping system [46]. In addition, research findings indicate that the release of autacoids (biological factors that function as local hormones) and prostaglandins can increase motility and electrolyte secretion in the small intestine [33]. In one experiment, *C. reflexa* caused attenuation of prostaglandin release [47], which may also contribute to its antidiarrheal action. However, the antidiarrheal potential of *C. reflexa* may be mediated by its previously reported anticholinergic or antihistaminic effects and the arrest of GIT motility, as well as some additional unidentified mechanisms.

The anticholinergic and antihistamine effects are responsible for the anti-secretory actions because the cholinergic and histaminergic receptors, when occupied by agonists, cAMP, are elevated at the intracellular level. This will then stimulate the enzyme kinase leading to greater protein synthesis acting as aquaporins, which increase secretion. The antagonist of these receptors, therefore, blocks these aquaporins syntheses and secretion is thus, stopped. Similarly, hypermotility is a characterized form of diarrhea that does not take into account the secretary causal factor. Pretreatment of animals with the tested plant material suppressed the movement of the charcoal plug in the gastrointestinal tract, indicating that *C. reflexa* inhibits GIT motility in a dose-dependent manner similar to loperamide. Loperamide has a direct effect on reducing stool volume and increasing viscosity by further reabsorption of water and electrolytes in the small intestine. Results of this study showed that *C. reflexa* inhibits gastrointestinal motility through its anticholinergic action. In addition, *C. reflexa* exhibits antibacterial, antifungal, and anthelmintic properties [48]. The bacterial toxin also stimulates G-coupled proteins, which, through intracellular signaling, increase aquaporins production and secretion. Therefore, the antibacterial effect stops the release of toxins. These properties of the plant can bring additional benefits to its antidiarrheal effect, including those of infectious diarrhea.

## 5 Conclusions

In summary, findings from this investigation suggest that the *C. reflexa* display remarkable antidiarrheal effect in different experimental models. Findings also showed that *C. reflexa* is an important antimotility and antisecretory herbal plant, contributing to its antidiarrheal potential. These findings may explain the medicinal use of *C. reflexa* as antidiarrheal medicinal plant in folk medicine, and provide a scientific basis to its traditional use as an antidiarrheal agent. However, more detailed studies are required to establish the safety, efficacy, and active constituents of this plant.

## Funding

None.

## Author’s contributions

SU, NM, MA, and OS, carried out the experimental part, AR supervised this work. SP, UF, and SA edited this paper. MS, SB, ERE, and MSM edited the final version of the manuscript. All authors read and approved this manuscript for finial submission.

## Ethics approval

All the protocols and procedures involving animals and their care were conducted as per the ethical guidelines approved by the Department of Pharmacy, University of Peshawar, Pakistan. Moreover, research was conducted in accordance with the internationally accepted principles for laboratory animal use and care.

## Consent to participate

Not Applicable.

## Availability of data and materials

Not Applicable.

## Declaration of Competing Interest

The authors declare that they have no known competing financial interests or personal relationships that could have appeared to influence the work reported in this paper.
